# Prevalence and Severity of Myocardial Perfusion Imaging Abnormalities in Inmate Subjects

**DOI:** 10.1371/journal.pone.0133360

**Published:** 2015-07-22

**Authors:** Roberta Assante, Emilia Zampella, Wanda Acampa, Carmela Nappi, Valeria Gaudieri, Nicola Frega, Davide D’Arienzo, Marianna Tuccillo, Pierpaolo Di Lorenzo, Claudio Buccelli, Mario Petretta, Alberto Cuocolo

**Affiliations:** 1 Department of Advanced Biomedical Sciences, University Federico II, Naples, Italy; 2 Institute of Biostructure and Bioimaging, National Council of Research, Naples, Italy; 3 Department of Translational Medical Sciences, University Federico II, Naples, Italy; University of Naples Federico II, ITALY

## Abstract

**Aim:**

We evaluated the prevalence and severity of myocardial perfusion abnormalities among inmates undergoing cardiac single-photon emission computed tomography. We also compared the results with those obtained in a cohort of non-inmates.

**Methods:**

Between January 2009 and December 2013, 2420 consecutive subjects (258 inmates and 2162 non-inmates) with suspected or known coronary artery disease underwent stress myocardial perfusion single-photon emission computed tomography (MPS) to our institution. The decision to submit inmates to MPS was taken by the physicians of the penal institutions or ordered by the court based on the survey of part. To account for differences in clinical characteristics between inmates and non-inmates, we created a propensity score-matched cohort considering clinical variables and stress type.

**Results:**

Before matching, inmates were younger and had higher prevalence of male gender, smoking, chest pain, and previous myocardial infarction or revascularization (all *p* < 0.001). After matching, all characteristics were comparable in 258 inmates and 258 non-inmates. The total amount of abnormal myocardium was similar in inmates and non-inmates before and after matching. Infarct size and severity were larger in inmates before (*p* < 0.001) and after (*p* < 0.01) matching and left ventricular ejection fraction was lower in inmates compared to non-inmates (*p* < 0.01).

**Conclusions:**

Detention is associated with larger infarct size compared to a general population of subjects referred to stress MPS also after matching for clinical variables and stress type. The similar prevalence of normal MPS in the matched cohort suggests that this imaging technique might be appropriate in inmates.

## Introduction

More than 10 million people are imprisoned worldwide and this number has increased by about a million in the past decade [1]. Prisoners bear a substantial burden of physical disorders relative to the general population and incarceration is associated with an increased risk of chronic diseases [2,3]. Many studies focused on prisoners and mental health, infectious diseases, including human immunodeficiency virus and hepatitis B and C infection, and cancer. Results from a survey in the United States have also shown higher age-adjusted rates of hypertension, diabetes, asthma, and arthritis in prisoners than in the general population [2]. Coronary artery disease (CAD) is the second most common cause of death in patients with history of incarceration, but the mechanisms of this increased risk are not still explained [4]. An increase in cardiovascular risk factors has been reported in patients with history of detainment, with a significantly elevated risk of future hypertension and left ventricular (LV) hypertrophy, as compared to general population [5]. The augmented prevalence of cardiovascular risk factors associated with incarceration may explain in part the increased risk of heart disease and death in prisoners [6]. Information about the risk and causes of death after release from prison could focus preventive efforts, improve transitional care, and guide policies to improve outcomes [4]. However, data about the prevalence of CAD in prisoners are still limited. This study evaluated the prevalence and severity of myocardial perfusion abnormalities among inmates undergoing cardiac single-photon emission computed tomography. We also compared the results with those obtained in a cohort of non-inmates.

## Materials and Methods

### Study population

**Fig 1:**
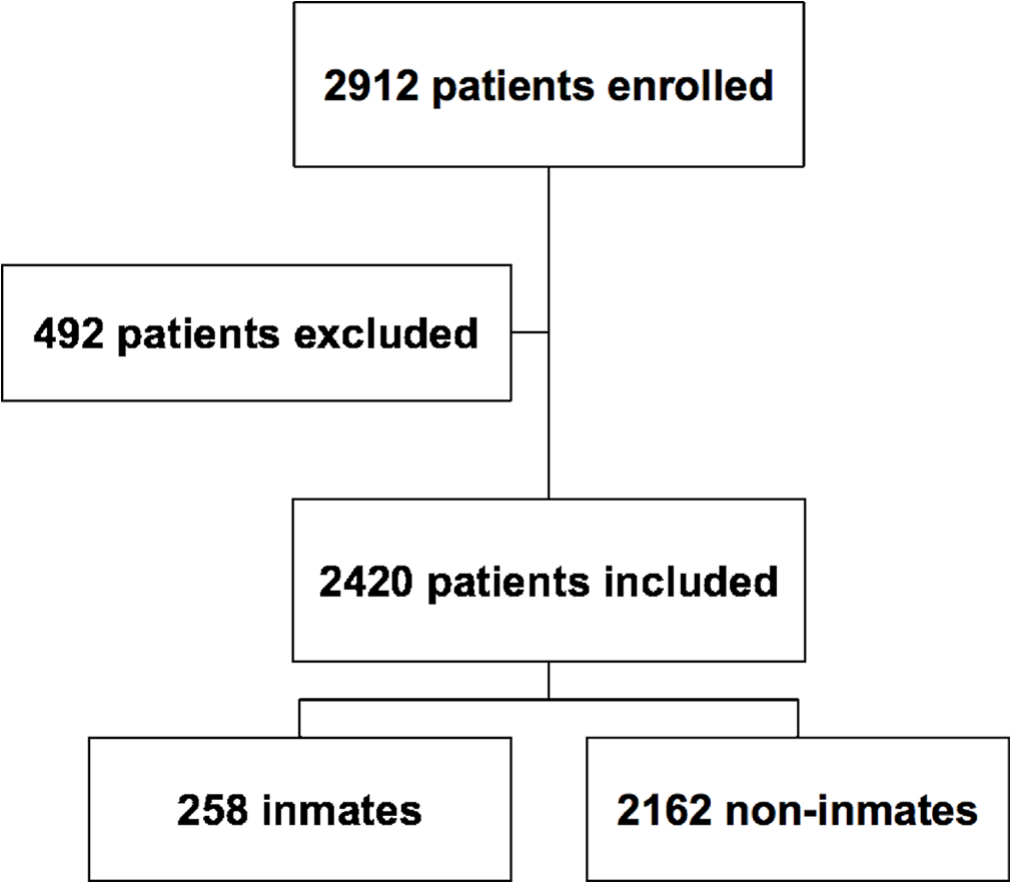
Flow-chart of patients selection. Between January 2009 to December 2013, 2912 consecutive subjects, with suspected or known CAD underwent stress gated MPS at our institution. Of these, 492 were excluded from study enrollment for recent acute coronary syndrome, stroke, or transient ischemic attack; uncompensated congestive heart failure; recent myocardial revascularization procedures. Of the remaining 2420 subjects, 258 were inmates.

Between January 2009 to December 2013, 2912 consecutive subjects, with suspected or known CAD underwent stress gated myocardial perfusion single-photon emission computed tomography (MPS) for the assessment of myocardial ischemia to our institution (Fig 1). Of these subjects, 492 were excluded from study enrollment for: 1) recent acute coronary syndrome, stroke, or transient ischemic attack (in the last 3 months); 2) uncompensated congestive heart failure (New York Heart Association class III or IV); 3) recent myocardial revascularization procedures (in the last 3 months). Of the remaining 2420 subjects, 258 were inmates from the 13 penal institutions located in Campania (South of Italy), an area that can take into custody 6085 prisoners [7]. The decision to submit the prisoners to MPS was taken by the physicians of the penal institutions or possibly ordered by the court based on the survey of part. As part of the baseline examination, clinical teams collected information on traditional cardiovascular risk factors, including age, gender, blood pressure, diabetes, smoking history, serum cholesterol, family and personal history of CAD, rest electrocardiography (ECG) characteristics, as well as the results of ECG stress testing [8]. The study was done in accordance with the current revision of the Declaration of Helsinki and applicable to national and local laws and regulations. The Ethics Committee of Federico II University Hospital Trust approved this observational study, the Department of Advanced Biomedical Sciences of the University of Naples Federico II collected the data and all patients provided written informed consent.

### Myocardial perfusion imaging

All subjects underwent same-day stress-rest ^99m^Tc sestamibi gated MPS by physical exercise or dipyridamole stress test, according to the recommendations of the European Association of Nuclear Medicine and European Society of Cardiology [9], as previously described in details [10]. Beta-blocking medications and calcium antagonists were withheld for 48 hours and long acting nitrates for 12 hours before testing. For subjects undergoing exercise test, symptom-limited treadmill standardized protocols were performed, with monitoring of heart rate and rhythm, blood pressure, and ECG. Test endpoints were horizontal or downsloping ST-segment depression ≥2 mm, ST-segment elevation ≥1 mm, moderate to severe angina, systolic blood pressure decrease ≥20 mm Hg, blood pressure ≥230/120 mm Hg, dizziness, or clinically important cardiac arrhythmia. The exercise stress test was considered submaximal if the subjects did not reach at least the 85% of maximal predicted heart rate in the absence of ECG ischemic changes and/or moderate to severe angina. For dipyridamole stress test, subjects were instructed to avoid products containing caffeine for 24 hours before the test. Dipyridamole was infused at dose of 0.142 mg × kg^-1^ × minute^-1^ intravenous over 4 minutes. A dose of 100 mg of aminophylline was administered intravenously in the event of chest pain or other symptoms, or after significant ST depression.

At peak exercise, or 4 min after completion of dipyridamole infusion, a bolus of 370 MBq of ^99m^Tc sestamibi was intravenously injected. Four hours after the conclusion of stress test, 1110 MBq of the tracer were injected at rest. Gated MPS imaging was performed 30 and 60 minutes after tracer injection for post-stress and rest study, respectively, using a dual-head rotating gamma camera (E.CAM, Siemens Medical Systems, Hoffman Estates, IL, USA) equipped with a low-energy, high-resolution collimator and connected with a dedicated computer system. No attenuation or scatter correction was used. After filtered back-projection, short-axis, vertical, and horizontal long-axis tomograms were generated.

An automated software program (e-soft, 2.5, QGS/QPS, Cedars-Sinai Medical Center, Los Angeles, CA) was used to calculate LV ejection fraction and the scores incorporating both the extent and severity of perfusion defects, using standardized segmentation of 17 myocardial regions [11]. Each myocardial segment was scored from normal (score = 0) to absent perfusion (score = 4). The summed stress score, representing the total myocardium abnormal, was obtained by adding the scores of the 17 segments of the stress images. A similar procedure was applied to the resting images to calculate the summed rest score, a measure of infarct size and severity. The summed difference score is the difference between the stress and rest scores and is taken to be an index of ischemic burden. MPS was considered abnormal when summed stress score was ≥3. Subjects with summed difference score ≥2 were defined as having stress-induced myocardial ischemia.

### Statistical analysis

Continuous variables were expressed as mean ± standard deviation and categorical data as percentages. Groups were compared using t-test, *χ* ^2^-test or Fisher’s exact test, as appropriate. A *p* value <0.05 was considered statistically significant. To account for differences in baseline characteristics between inmates and non-inmates, we also created a propensity score-matched cohort. The propensity score (logit model) was calculated for each individual based on the baseline clinical variables (age, sex, dyslipidemia, smoking, hypertension, family history of CAD, chest pain symptoms, history of myocardial infarction, or revascularization procedures) and stress type. A 1-to-1 matched analysis without replacement was performed on the basis of the estimated propensity score of each patient [12]. To perform the matching and to select the final data set for analysis, the nearest available Mahalanobis metric matching method with caliper size specification (0.25 × standard deviation of propensity score) was used. After propensity score matching, baseline characteristics were compared. In addition, we assessed the success of propensity score matching using standardized differences [13].

## Results

### Non-matched cohort

Demographic data and clinical characteristics in inmate and non-inmate subjects before propensity score matching are shown in Table 1. Inmates were younger and had a higher prevalence of male gender, smoking, hypertension, chest pain, and previous myocardial infarction or coronary revascularization. A lower percentage of inmates (*p* < 0.001) were referred for a pharmacologic stress test. Among subjects undergoing exercise testing, 99 inmates (50%) and 344 non-inmates (29%) performed a submaximal stress test (*p* < 0.001). The achieved metabolic equivalent threshold was 8.73 ± 2.7 in inmates and 9.44 ± 2.7 in non-inmates (*p* < 0.005). ECG ischemic changes during exercise stress test occurred in 3% of inmates and in 8% of non-inmates (*p* <0.05). Angina symptoms requiring exercise stress test interruption occurred in 3% of inmates and in 1% of non-inmates (*p* < 0.05).

**Table 1:** Demographic data and clinical characteristics by inmate status before propensity score matching.

	Inmates (n = 258)	Non-inmates (n = 2162)	*p value*
Age (y)	53 ± 10	63 ± 11	<0.001
Male gender	256 (99%)	1423 (66%)	<0.001
Dyslipidemia	166 (64%)	1345 (62%)	0.50
Diabetes	95 (37%)	737 (34%)	0.38
Smoking	155 (60%)	797 (37%)	<0.001
Hypertension	185 (72%)	1741 (80%)	<0.001
Family history of CAD	117 (45%)	1105 (51%)	0.08
Chest pain symptoms	146 (57%)	758 (35%)	<0.001
Prior myocardial infarction	141 (55%)	644 (30%)	<0.001
Prior revascularization	113 (44%)	590 (27%)	<0.001
Dipyridamole stress test	61 (24%)	978 (45%)	<0.001
Exercise stress test	197 (76%)	1184 (55%)	<0.001
Submaximal exercise stress test	99 (50%)	344 (29%)	<0.001

Values are expressed as mean value ± standard deviation or as number (percentage) of subjects. CAD: coronary artery disease.

**Fig 2:**
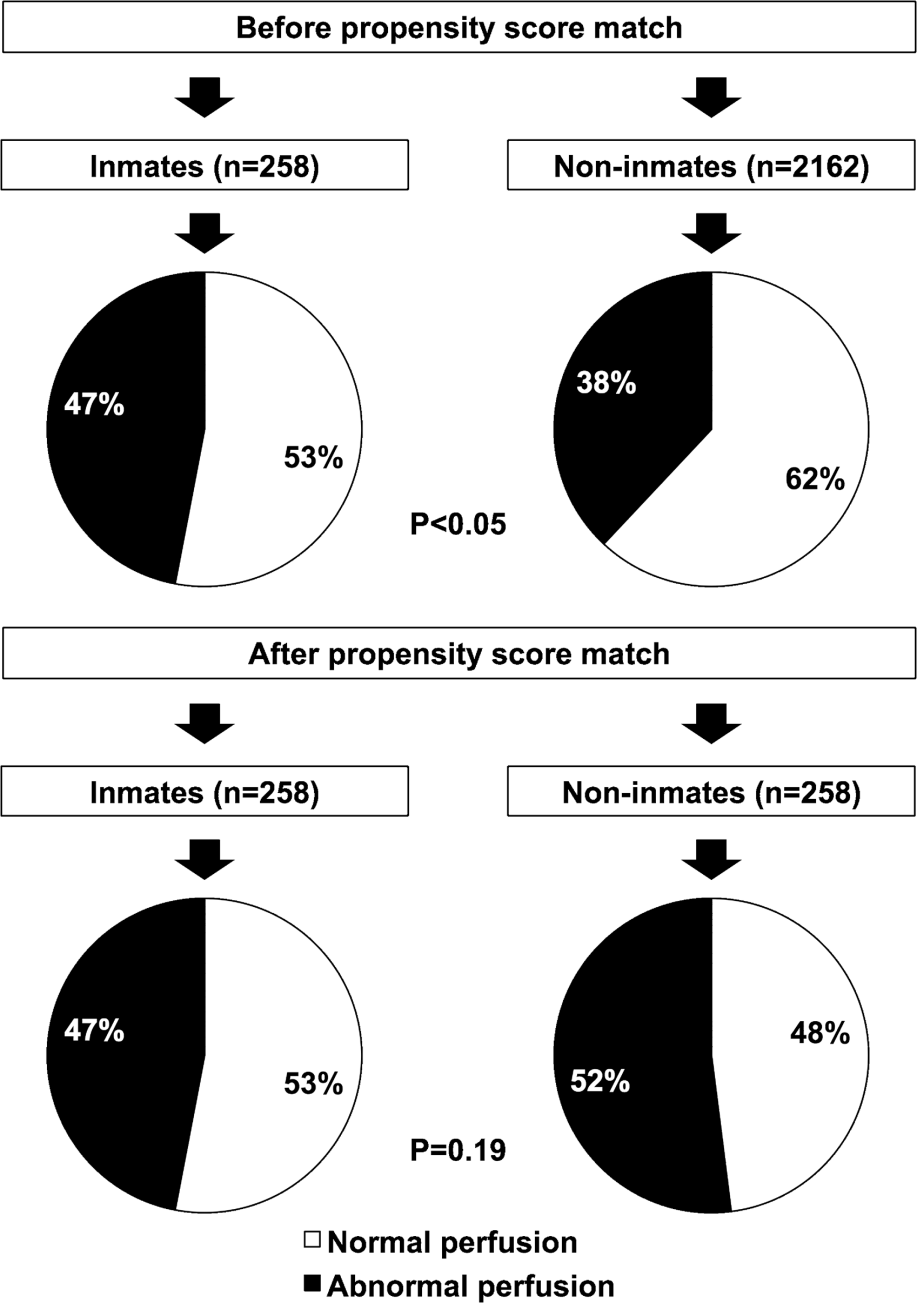
Myocardial perfusion results in inmates and non-inmates. Before propensity score matching myocardial perfusion was abnormal in a higher percentage of inmates compared to non-inmates (*p* < 0.05), while after matching the percentage of abnormal perfusion was similar in inmates and non-inmates (*p* = 0.19).

MPS results are reported in Table 2. The summed stress score, representing the total myocardium abnormal, was comparable in inmates and non-inmates, while summed rest score value, an index of infarct size and severity, was higher (*p* < 0.001) in inmates. Rest and post-stress LV ejection fraction values were significantly lower in inmates as compared to non-inmates (both *p* < 0.001). On a subject-based analysis, myocardial perfusion was abnormal in 119 (47%) inmates and 832 (38%) non-inmates (*p* < 0.05) (Fig 2). Noteworthy, a higher percentage of inmates had evidence of myocardial necrosis compared to non-inmates (36% vs. 27% *p* < 0.05), while no difference was detectable in the prevalence of stress-induced ischemia.

**Table 2:** MPS results by inmate status before propensity score matching.

	Inmates (n = 258)	Non-inmates (n = 2162)	*p value*
Summed stress score	5.6 ± 7	5.5 ± 7	0.83
Summed rest score	4.5 ± 7	2.8 ± 6	<0.001
Summed difference score	1.1 ± 2	1.3 ± 2	0.13
Rest LVEF (%)	46 ± 12	52 ± 16	<0.0001
Post-stress LVEF (%)	51 ± 13	61 ± 17	<0.0001

Values are expressed as mean value ± standard deviation. LVEF: left ventricular ejection fraction.

### Matched cohort

After propensity score matching, 258 well-matched pairs were identified. The demographic and clinical characteristics were similar for the inmates and non-inmates (Table 3). Pharmacologic stress test was performed in 61 inmates and 60 non-inmates. Exercise stress testing was submaximal in 50% of inmates and 29% of non-inmates (*p* < 0.001). The achieved metabolic equivalent threshold was 8.73 ± 2.7 in inmates and 10.4 ± 2.8 in non-inmates (*p* < 0.001). ECG ischemic changes during exercise stress test occurred in 3% of inmates and in 8% of non-inmates (*p* <0.05). Angina symptoms requiring exercise stress test interruption occurred in 3% of inmates and in 1% of non-inmates (*p* < 0.05).

**Table 3:** Demographic data and clinical characteristics by inmate status after propensity score matching.

	Inmates (n = 258)	Non-inmates (n = 258)	*p value*
Age (y)	53 ± 10	52 ± 11	0.73
Male gender	256 (99%)	256 (99%)	1.00
Dyslipidemia	166 (64%)	170 (66%)	0.71
Diabetes	95 (37%)	82 (32%)	0.20
Smoking	155 (60%)	147 (57%)	0.48
Hypertension	185 (72%)	182 (70%)	0.77
Family history of CAD	117 (45%)	120 (46%)	0.79
Chest pain symptoms	146 (57%)	138 (53%)	0.48
Prior myocardial infarction	141 (55%)	137 (53%)	0.72
Prior revascularization	113 (44%)	117 (45%)	0.72
Dipyridamole stress test	61 (24%)	60 (24%)	1.00
Exercise stress test	197 (76%)	198 (77%)	0.98
Submaximal exercise stress test	99 (50%)	57 (29%)	<0.001

Values are expressed as mean value ± standard deviation or as number (percentage) of subjects. CAD: coronary artery disease.

MPS results are reported in Table 4. The summed rest score was higher in inmates (*p* < 0.05), while summed difference score was higher in non-inmates (*p* < 0.005). Rest LV ejection fraction was lower in inmates compared to non-inmates (*p* < 0.01). On a subject-based analysis, both the percentage of abnormal MPS (47% and 52%, *p* = 0.19) and the percentage of subjects with myocardial necrosis (36% and 31%, *p* = 0.22) were similar in inmates and non-inmates. On the contrary, the percentage of subjects with stress-induced ischemia was higher in non-inmates compared to inmates (38% vs. 24%, *p* < 0.005). MPS results stratified by stress type are reported in Table 5. In patients undergoing dipyridamole stress test MPS data were comparable in inmates and non-inmates. Conversely, after exercise stress test summed difference score was higher in non-inmates compared to inmates (2.1 ± 3 vs. 1.0 ± 2; *p* < 0.001).

**Table 4:** MPS results by inmate status after propensity score matching.

	Inmates (n = 258)	Non-inmates (n = 258)	*p* value
Summed stress score	5.6 ± 7	5.3 ± 7	0.63
Summed rest score	4.5 ± 7	3.2 ± 7	<0.05
Summed difference score	1.1 ± 2	1.8 ± 3	<0.005
Rest LVEF (%)	46 ± 12	49 ± 13	<0.01
Post-stress LVEF (%)	51 ± 13	53 ± 14	0.09

Values are expressed as mean value ± standard deviation. LVEF: left ventricular ejection fraction.

**Table 5:** MPS results by inmate status and stress test type after propensity score matching.

	Dipyridamole stress test			Exercise stress test		
	Inmates (n = 61)	Non-inmates (n = 60)	*p value*	Inmates (n = 197)	Non-inmates (n = 198)	*p value*
Summed stress score	5.8 ± 7	5.1 ± 7	0.58	5.1 ± 6	6.0 ± 7	0.17
Summed rest score	4.7 ± 7	3.0 ± 7	0.18	4.0 ± 6	3.8 ± 7	0.76
Summed difference score	1.2 ± 2	1.6 ± 2	0.27	1.0 ± 2	2.1 ± 3	<0.001
Rest LVEF (%)	51 ± 12	55 ± 13	0.08	51 ± 13	48 ± 13	<0.05
Post-stress LVEF (%)	46 ± 12	50 ± 12	0.07	47 ± 13	45 ± 13	0.12

Values are expressed as mean value ± standard deviation. LVEF: left ventricular ejection fraction.

## Discussion

The major finding of our study is that, compared to a general population of subjects referred to stress MPS, inmates more frequently have evidence of prior myocardial infarction, probably related to a higher prevalence of cardiovascular risk factors. Inmates show larger infarct size and severity also after taking into account clinical variables and stress type by propensity score matching.

Detention is associated with an increased risk of poor medical outcomes and mortality compared with the general population, due to circumstances before and during incarceration [14]. Inmates often come from disadvantaged backgrounds and have low levels of education. Moreover, a higher prevalence of smoking and drug abuse in inmates before and during incarceration has been reported [15,16]. The high rates of intravenous drug use in prisoners also leads to increased alcohol misuse and smoking. These behaviors in turn raise the risk of cardiovascular disease, a heavy burden also detectable in subjects having a family member incarcerated [17].

Ceelen et al. [18] found that heart diseases were among the most common somatic diagnoses for forensic medical service. The authors also found that, after correction for age and gender, detainees appeared to suffer more often than the general population from hypertension and serious heart diseases. Ex inmates are almost twice as likely to be diagnosed with heart problems [19]. On average, inmate patients with heart disease stay in the hospital longer and receive treatment sooner compared to non-inmate patients, indicating that inmates do not receive poorer quality of care compared to non-inmate patients [20].

At this time no studies have explored the prevalence and severity of myocardial perfusion defects in an inmate population during incarceration. Thus, in the present study we compared stress MPS results between inmates and non-inmates subjects. We found that inmates have a higher prevalence of cardiovascular risk factors and prior myocardial infarction. Similar findings were reported by Richmond et al. [21], who found that 39% of the male prison population had three or more cardiovascular risk factors compared to only 10% of disadvantaged men of similar age in the community. Wang et al. [5] examined the association of prior incarceration with incident hypertension, diabetes, and dyslipidemia in young adults and they found that incarceration was associated with future hypertension and LV hypertrophy. Arries et al. [22] found that smoking, physical inactivity, obesity, and hypertension were common CAD risk factors found in prisoners. Data from Medicare administrative claims indicate that transitions between correctional facilities and the community may be a high-risk period and have potential implications for both the correctional health care and community health care systems [23]. Thus, identifying prisoners at high risk of CAD could improve the development of prevention and treatment strategies specifically directed to this population. To overcome potential bias due to possible confounding, such as concomitant cardiovascular risk factors, we also performed a propensity score-matched analysis of a cohort of inmates and non-inmates. After matching for the clinical variables and stress type, inmates with suspected or known CAD referred to stress MPS showed a larger infarct size and severity compared to non-inmates. Unmeasured variables related to lifestyle, environmental and socio-economic factors, detention related stress, and the likely drug addiction might explain the worse outcome of ischemic heart disease in inmates. As a result of increasing number of prisoners and CAD epidemics, it would be necessary to improve prison health-care services, through better integration between prison and public health systems and continuity care for individuals transitioning to community-based health care after release from prison [24,25]. This improvement can result in more appropriated treatment and costly use of health care.

It should be considered that MPS is expensive and that to be performed the inmates must be transferred to another facility, with inherent security problems. However, the similar prevalence of normal MPS in the matched cohort of inmates and non-inmates suggests that this imaging technique is appropriate also during incarceration. From our study it also emerged that the prevalence of stress-induced myocardial ischemia in non-inmates is higher as compared to inmates. This result is not surprisingly considering the higher prevalence of submaximal exercise test in inmates compared to non-inmates. Of note, with dipyridamole stress-induced ischemia was comparable in inmates and non-inmates. Therefore, a pharmacological stress test should be preferred in inmates to make the procedure more reliable limiting the number of non-diagnostic test.

This study has some limitations. Although propensity scores are widely used this approach can balance observed baseline covariates between groups but not unmeasured confounders. Another limitation is the inherent selection bias in the referring process for prisoners and non-prisoners, including specific legal aspects governing detention that may differ among countries.

## Conclusions

Detention is associated with a larger infarct size and severity as compared to a general population of subjects referred to stress MPS also after matching for clinical variables and stress type. The similar prevalence of normal myocardial perfusion in the propensity score-matched cohort suggests that this imaging technique might be appropriate in inmates. Finally, in the absence of contraindications pharmacological stress test should be recommended in inmates undergoing MPS to limit the number of non-diagnostic test.

## Supporting Information

S1 Dataset Non-matched cohort. Individual demographic data, clinical characteristics and MPS results in inmate and non-inmate subjects before propensity score matching.
(XLS)

S2 Dataset Matched cohort. Individual demographic data, clinical characteristics and MPS results in inmate and non-inmate subjects after propensity score matching.
(XLS)
